# Chromosome‐level genome and methylome of vine tea suggest roles for tandem duplication and CHH hypomethylation in high dihydromyricetin accumulation

**DOI:** 10.1002/imo2.70129

**Published:** 2026-08-06

**Authors:** Yingmei Wu, Hanrui Hu, Qing Ye, Xueqing Chen, Miyuan Tian, Jing Li, Li Lu, Yifei Liu, Yi Shang, Yanling Li, Xingyao Xiong, Junbo Gou

**Affiliations:** ^1^ Hubei Shizhen Laboratory, Hubei Key Laboratory of Resources and Chemistry of Chinese Medicine, College of Pharmacy Hubei University of Chinese Medicine Wuhan China; ^2^ Shenzhen Huayi Xinchuang Biotechnology Co., Ltd. Shenzhen China; ^3^ Laifeng County Rural Investment and Construction Co., Ltd. Enshi China; ^4^ State Key Laboratory of Plant Diversity and Specialty Crops, Wuhan Botanical Garden Chinese Academy of Sciences Wuhan China; ^5^ State Key Laboratory of Hybrid Rice, School of Pharmaceutical Sciences Wuhan University Wuhan China; ^6^ Engineering Research Center for Valorization of Unique Bio‐Resources in Yunnan, Ministry of Education Yunnan Normal University Kunming China; ^7^ College of Horticulture Hunan Agricultural University Changsha China; ^8^ Shenzhen Branch, Guangdong Laboratory for Lingnan Modern Agriculture, Shenzhen Key Laboratory of Agricultural Synthetic Biology, Agricultural Genomics Institute at Shenzhen Chinese Academy of Agricultural Sciences Shenzhen China

## Abstract

A chromosome‐level genome assembly and single‐base resolution methylome of vine tea (*Nekemias grossedentata*) reveal that lineage‐specific tandem duplications of dihydromyricetin (DHM) biosynthetic genes (*NgCHS* and *NgF3′5′ H*) have expanded their copy numbers, providing the genetic basis for high DHM flux. Tissue‐specific CHH hypomethylation in young leaves, associated with downregulation of RNA‐directed DNA methylation (RdDM)‐related methyltransferases and upregulation of the demethylase NgDML1, correlates with the selective high expression of these duplicated genes, while competing pathway branches (e.g., STS for stilbene synthase, HCT for shikimate hydroxycinnamoyl transferase, DFR for dihydroflavonol 4‐reductase) maintain hypermethylation and low expression. Candidate v‐myb avian myeloblastosis viral oncogene homolog (MYB) and basic helix‐loop‐helix (bHLH) transcription factors co‐expressed with DHM pathway genes are identified. These findings suggest a “duplication‐associated activation” model integrating genomic, epigenomic, and transcriptomic layers for high DHM accumulation.

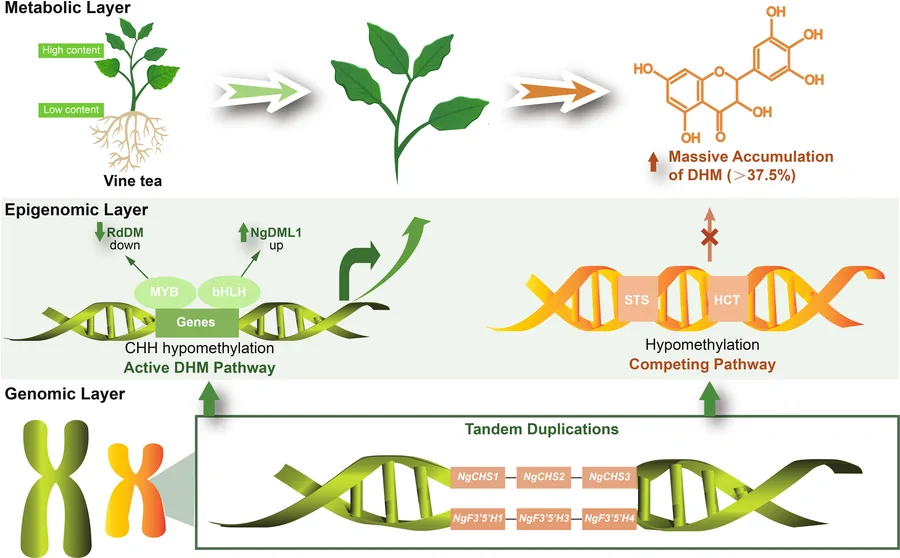

To the Editor,

Vine tea (*Nekemias grossedentata*) is a traditional medicinal beverage from southern China that hyperaccumulates the bioactive flavonoid dihydromyricetin (DHM), which comprises >37.5% of the dry weight in young leaves [1, 2]. DHM exhibits antioxidant, anti‐aging, anti‐inflammatory, and hepatoprotective activities [3, 4]. Recent studies have provided valuable genome assemblies and identified cultivar‐specific structural variations in biosynthetic genes [5, 6]; however, the regulatory mechanisms underlying tissue‐specific DHM hyperaccumulation, particularly the potential roles of DNA methylation and the evolutionary context of gene duplication, remain largely unexplored.

Here, we present a chromosome‐level genome assembly of *N. grossedentata* (scaffold N50 = 31.87 Mb, BUSCO = 97.4%; 98.55% of sequences anchored to 20 pseudochromosomes) together with the first whole‐genome DNA methylome for this species at single‐base resolution. Integrative analysis reveals three key findings. First, lineage‐specific tandem duplications of DHM biosynthetic genes, especially *NgCHS* and *NgF3′5′ H*, have expanded their copy numbers and enabled their coordinated high expression in young leaves, providing the genetic basis for elevated DHM flux. Second, tissue‐specific CHH hypomethylation in young leaves is strongly associated with the high expression of duplicated DHM biosynthetic genes and the hypermethylation of competing pathway branches; this hypomethylated state correlates with downregulation of RNA‐directed DNA Methylation (RdDM)‐related methyltransferases and upregulation of the demethylase NgDML1. Third, we identify candidate v‐myb avian myeloblastosis viral oncogene homolog (MYB) and Basic Helix‐Loop‐Helix (bHLH) transcription factors whose expression correlates with DHM pathway genes.

Together, our findings suggest that tandem duplication and CHH hypomethylation correlate with high DHM accumulation, leading to a testable “duplication‐associated activation” hypothesis. Unlike previous genomic studies [5, 6], our work reveals that 96.4% of core DHM genes are organized as tandem duplications and provides the first methylome for this species, adding an epigenetic layer.

## 1 A CHROMOSOME‐LEVEL GENOME ASSEMBLY REVEALS EXTENSIVE TANDEM DUPLICATION OF DHM BIOSYNTHETIC GENES

To investigate the genetic basis of high DHM accumulation, we generated a chromosome‐level genome assembly of *N. grossedentata* using PacBio HiFi and Hi‐C sequencing, yielding a 620 Mb assembly anchored to 20 pseudochromosomes (scaffold N50 = 31.87 Mb; 98.55% of sequences anchored) (Figures 1A,B and S1, Tables S1–S9). Phylogenomic analysis using 116 single‐copy orthologs indicated that Vitaceae diverged from other angiosperms approximately 105.2 million years ago (Mya). Within Vitaceae, the tribe Cisseae diverged first (~57.9 Mya), followed by the divergence of Ampelopsideae (represented by *N. grossedentata*) and Viteae (including *Vitis vinifera*) ~45.4 Mya (Figure 1C and Table S10). This phylogenetic framework supports Cisseae as the earliest‐diverging lineage within Vitaceae and provides evolutionary context for further resolving the positions of other tribes, such as Cayratieae and Parthenocisseae [7, 8, 9, 10] and metabolic specialization in this lineage.

**Figure 1:**
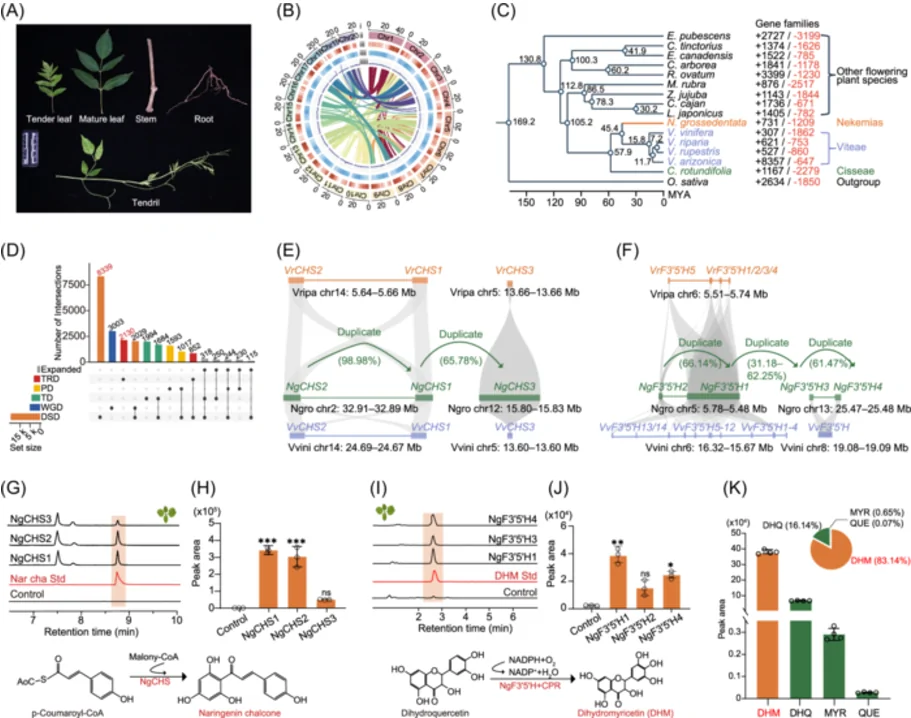
Genomic and epigenomic features associated with high DHM accumulation in vine tea. (A) Photographs of different *N. grossedentata* tissues: tender leaf, mature leaf, stem, root, and tendril. Scale bar in the root panel (5 cm) applies to all photographs. (B) Circos plot of the 20 pseudochromosomes. Tracks from outer to inner: chromosome ideogram (Mb), gene density, GC content, LTR retrotransposon density, and DNA transposon density. (C) Phylogenetic tree of *N. grossedentata* and related species within Vitaceae and other angiosperms, constructed using 116 single‐copy orthologs. Divergence times (million years ago, Mya) are indicated at the nodes. Black and red numbers indicate expanded and contracted gene families, respectively. (D) Classification of gene duplication modes in the vine tea genome. (E) Genomic localization and synteny analysis of the three *NgCHS* genes in *N. grossedentata* compared to *Vitis vinifera* and *Vitis riparia*. Arrows indicate gene orientation. (F) Genomic localization and synteny analysis of the four *NgF3′5′ H* genes compared to *V. vinifera* and *V. riparia*, illustrating two independent tandem arrays on chromosomes 5 and 13. (G) Functional analysis of NgCHS proteins in *Nicotiana benthamiana*. Representative UHPLC‐Q‐TOF‐MS chromatograms showing naringenin chalcone production. (H) Quantitative analysis of naringenin chalcone production in *N. benthamiana* leaves expressing *NgCHS1*, *NgCHS2*, or *NgCHS3*. Control, empty vector control. Data are mean ± SD (*n* = 3); one‐way ANOVA with Tukey's test. ***p < 0.001 compared with control. (I) Functional analysis of three of the four NgF3′5′ H proteins. Representative chromatograms showing DHM production in *N. benthamiana* leaves co‐expressing *NgF3′5′ H* and *LjCPR*. (J) Quantitative analysis of DHM production in *N. benthamiana* leaves expressing *NgF3′5′ H1*, *NgF3′5′ H3*, or *NgF3′5′ H4*. Data are mean ± SD (*n* = 3); one‐way ANOVA with Tukey's test. ***p < 0.001 compared with control. (K) HPLC quantification of major flavonoids in young leaves, showing DHM as the dominant compound. DSD, dispersed duplication; DHM, dihydromyricetin; DHQ, dihydroquercetin; HPLC, high performance liquid chromatography; LTR, long terminal repeat; MYA, million years ago; MYR, myricetin; Ng, *N. grossedentata*; PD, proximal duplication; QUE, quercetin; TD, tandem duplication; TRD, transposed duplication; UHPLC‐Q‐TOF‐MS, ultra‐high performance liquid chromatography‐quadrupole time‐of‐flight mass spectrometry; Vr, *V. riparia*; Vv, *V. vinifera*; WGD, whole‑genome duplication.

Tandem duplication (TD) accounts for 17.25% of duplicated genes in the vine tea genome (Figure 1D and Table S11). Expanded gene families were significantly enriched in phenylpropanoid and flavonoid biosynthesis pathways (Table S12). We annotated 101 genes encoding nine key enzymes in the phenylpropanoid‐flavonoid pathway [11] (*e.g*., PAL, C4H, 4CL, CHS, CHI, F3H, F3′5′ H, F3′ H, and FLS), alongside 82 genes from competing branches (*e.g*., STS, stilbene synthase; DFR, dihydroflavonol 4‐reductase; HCT, shikimate hydroxycinnamoyl transferase) (Figure S2 and Tables S13–S15). Comparative genomics across Vitaceae revealed no overall expansion of these families in vine tea (Figure S2 and Table S16). Strikingly, 96.4% (27/28) of core DHM biosynthetic genes and their paralogs are organized in tandem arrays (Figure S3), effectively increasing the copy number. Other duplication modes (DSD, WGD, PD, TRD) also contributed to copy number gains (Figure S4 and Table S17). For example, *NgCHS1* and *NgCHS2* constitute a recent tandem duplication on chromosome 2 (98.98% identity), while *NgCHS3* on chromosome 12 originated from an older duplication event (~35.90–57.48 Mya) (Figure 1E, Tables S18 and S19). Similarly, the four *NgF3′5′ H* genes are arranged as two independent tandem pairs on chromosomes 5 and 13, with the *NgF3′5′ H3‐H4* pair originating from a dispersed duplication ~25.47–39.37 Mya (Figure 1F, Tables S20 and S21). Genome‐wide profiling identified 415 CYP450 and 179 *2‐ODD* genes, including four *CYP75A* (*NgF3′5′ H1‐4*), one *CYP75B* (*NgF3′ H*), and two *2‐ODD* (*NgF3H1‐2*) genes directly implicated in DHM hydroxylation (Tables S22–S24). Heterologous expression in *Nicotiana benthamiana* confirmed that all tested NgCHS1/2/3 and NgF3′5′ H1/3/4 proteins are catalytically active, producing naringenin chalcone (Figure 1G,H) and DHM (Figure 1I,J), respectively. Notably, while Vitis species contain both CHS and STS families, vine tea has not expanded STS genes, potentially directing carbon flux toward flavonoids rather than stilbenes. A recent pan‐genome study identified structural variations in *NgF3′5′ H* associated with inter‐cultivar DHM differences [6]; our findings complement that work by revealing the tandem genomic architecture enabling such variation, while providing the first methylome for this species.

In young leaves, these duplicated genes are highly expressed. DHM is the dominant flavonoid in young leaves (Figures 1K and S5, Table S25). The combined expression of *NgCHS1‐3* was > 21‐fold higher than that of competing branch genes such as *NgSTS*, and *NgF3′5′ H3* reached an FPKM of 1020.76 (Figure S6). This expression pattern suggests that tandem duplication has increased the copy number of functional enzymes and enabled coordinated, leaf‐specific overexpression. Together, these features establish a metabolic architecture that directs precursors toward high‐flux DHM production.

## 2 TISSUE‐SPECIFIC CHH HYPOMETHYLATION CORRELATES WITH ACTIVATION OF DHM PATHWAY GENES

A key distinction of our study from recent genomic works [5, 6] is the first whole‐genome DNA methylome for *N. grossedentata*. We performed whole‐genome bisulfite sequencing (WGBS) on roots, stems, and young leaves, producing single‐base resolution methylation maps (Figure 2A). Global analysis revealed that CG methylation was highest (41.84%–58.64%), followed by CHG (30.21%–39.21%), while CHH methylation remained consistently low across all tissues (9.35%–12.39%) (Figure 2B and Table S26). Notably, CHH methylation was significantly reduced in young leaves (9.35%) compared to roots (12.39%) and stems (12.14%), suggesting a potential role for CHH dynamics in leaf‐specific metabolic regulation. Methylation patterns were similar across tissues: within gene bodies, methylation was lowest near transcription start sites and increased toward transcription end sites, while TE regions showed elevated methylation, particularly in CHG and CHH contexts. CHH methylation was consistently lower in leaves across both genes and TEs (Figure S7 and Tables S27–S32).

**Figure 2:**
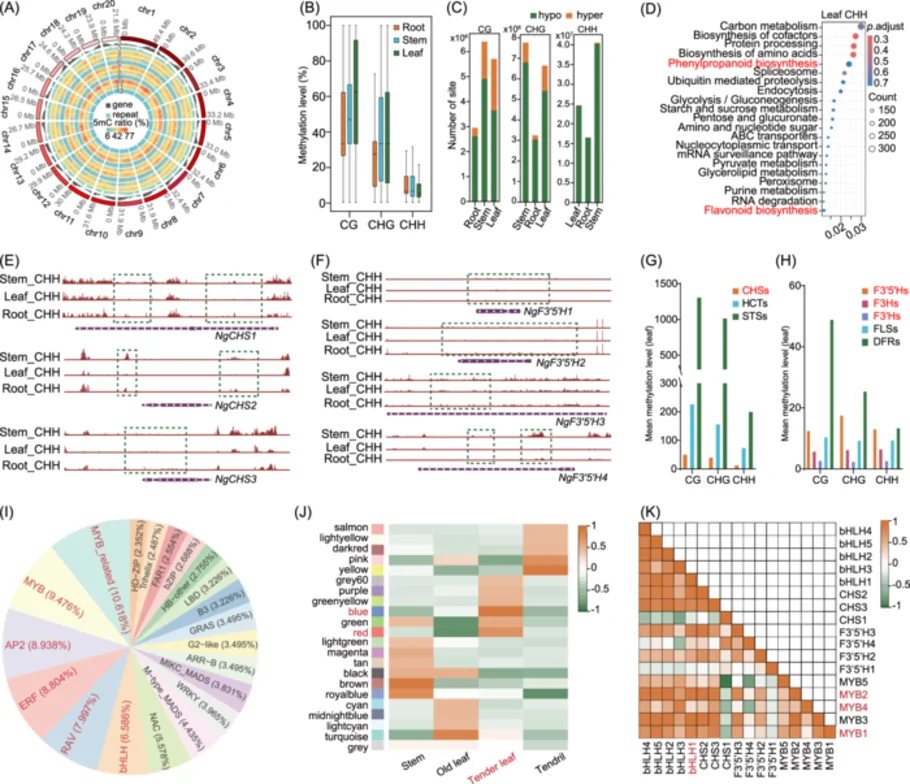
Tissue‐specific CHH hypomethylation correlates with DHM pathway activation and identifies candidate regulators. (A) Chromosomal landscape of DNA methylation levels across the 20 pseudochromosomes of *Nekemias grossedentata*. From the outermost to the innermost track, the tracks represent: 1, chromosome ideograms, with color intensity indicating chromosome size (darker colors represent longer chromosomes); 2, gene density; 3, transposable element (TE) density; 4, CpG methylation level in root; 5, CHG methylation level in root; 6, CHH methylation level in root; 7, CpG methylation level in stem; 8, CHG methylation level in stem; 9, CHH methylation level in stem; 10, CpG methylation level in young leaf; 11, CHG methylation level in young leaf; and 12, CHH methylation level in young leaf. Each chromosome was divided into non‐overlapping 1‐Mb bins to calculate gene density, TE density, and DNA methylation levels. The numbers shown on the outermost track indicate genomic positions along the corresponding chromosomes or chromosome lengths, with all values expressed in megabases (Mb). For the methylation tracks, the color scale from blue to red represents low to high methylation levels, respectively. (B) Global DNA methylation levels in CG, CHG, and CHH contexts across root, stem, and young leaf tissues (box plots, *n* = 3 biological replicates). Young leaves show pronounced CHH hypomethylation compared to roots and stems. (C) Number of hyper‐ and hypomethylated differentially methylated regions (DMRs) in root, stem, and young leaf tissues across CG, CHG, and CHH contexts. CHH hypomethylation bias is most pronounced in young leaves. (D) KEGG enrichment analysis of genes associated with CHH‐hypomethylated DMRs in young leaves. Significance is based on Benjamini‐Hochberg adjusted *p*‐values (*p* < 0.05). (E and F) Integrative Genomics Viewer (IGV) tracks showing CHH methylation levels in young leaves versus roots for key DHM biosynthetic genes: (E) the *NgCHS1*–*NgCHS3* locus; (F) the *NgF3′5′ H1*–*NgF3′5′ H4* loci. Tracks are labeled “Root” and “Young leaf.” The green dashed boxes highlight genomic regions where CHH methylation is markedly lower in young leaves compared to roots and stems. The purple bars represent gene structures, and the white arrows indicate the transcriptional direction of the genes. The displayed region encompasses the entire gene locus, from the transcription start site to the transcription termination site. Purple rectangles of different widths represent distinct gene structure regions: wider rectangles indicate exons, whereas narrower regions represent introns or untranslated regions (UTRs). (G) Mean DNA methylation levels (CG, CHG, and CHH contexts) in young leaves of *NgCHS* genes versus competing branch genes *NgHCTs* and *NgSTSs*. (H) Mean DNA methylation levels in young leaves for key DHM biosynthetic genes (*NgF3′5′Hs*, *NgF3Hs*, *NgF3′Hs*) versus competing downstream genes *NgFLSs* and *NgDFRs*. (I) Distribution of transcription factor families in the *N. grossedentata* genome. Pie chart showing the relative abundance of transcription factor (TF) families among the 1252 identified TFs. The most abundant families are MYB/MYB‐related (20.09%; MYB 9.48% + MYB_related 10.92%), AP2/ERF (17.74%; AP2 8.94% + ERF 8.80%), RAV (7.99%), and bHLH (6.59%). (J) Module‐trait association analysis showing the correlation between WGCNA‑identified co‑expression modules and different tissue types; the Red module (correlation = 0.8, *p* < 0.005) and Blue module (correlation = 0.97, *p* < 0.001) exhibited the strongest positive correlation with tender leaf. (K) Pearson correlation heatmap of shortlisted transcription factor candidates with key DHM biosynthetic genes; three MYB candidates (NgMYB1, NgMYB2, NgMYB4) and one bHLH candidate (NgbHLH1) exhibited strong positive correlations (*r* > 0.8) with multiple *NgCHS* and *NgF3′5′ H* genes. AP2, apetala2; bHLH, basic helix‐loop‐helix; CHS, chalcone synthase; DFR, dihydroflavonol 4‐reductase; DMR, differentially methylated region; ERF, ethylene response factor; F3′5′ H, flavonoid 3′,5′‐hydroxylase; F3H, flavanone 3‐hydroxylase; F3′ H, flavonoid 3′‐hydroxylase; FLS, flavonol synthase; HCT, shikimate hydroxycinnamoyl transferase; IGV, integrative genomics viewer; KEGG, kyoto encyclopedia of genes and genomes; MYB, v‐myb avian myeloblastosis viral oncogene homolog; NAC, nam ataf1/2 cuc2; RAV, related to abi/vp1; STS, stilbene synthase; TE, transposable element; TF, transcription factor; WGCNA, weighted gene co‐expression network analysis.

Differential methylation analysis identified 24.7 million CHH DMRs in young leaves, of which 99.57% were hypomethylated relative to other tissues, a ~232‐fold bias, far more pronounced than in CG or CHG contexts (Figure 2C and Table S33). This indicates that CHH hypomethylation is the most prominent epigenetic signature distinguishing young leaves. CHH hypo‐DMRs were predominantly enriched in intergenic regions (~63%) and promoters (~11%), with limited overlap across CG, CHG, and CHH contexts (<2%), indicating largely independent regulation (Figures S8 and S9, Tables S34 and S35). KEGG enrichment of genes associated with these CHH hypo‐DMRs showed significant enrichment in the flavonoid biosynthesis pathway (*p* < 0.001) and phenylpropanoid pathway (Figures 2D and S10, Tables S36–S44), directly linking this epigenetic feature to DHM metabolism [12, 13].

We next examined methylation patterns of key DHM pathway genes (Figure S11). In young leaves, *NgCHS1‐3*, *NgF3′5′ H1‐4*, and *NgF3H1‐2*, showed pronounced CHH hypomethylation in their promoter and genic regions (Figures 2E,F and S12–S16, Tables S45–S53). By contrast, competing branch genes, including *NgHCTs* (lignin pathway), *NgSTSs* (stilbene pathway), and *NgDFRs* (anthocyanin branch), maintained substantially higher CHH methylation (Figures S17–S24). Total CHH methylation of *NgCHS1‐3* was 16.82, whereas *NgSTSs* showed 204.53, a ~12‐fold difference (Figure 2G). Similarly, *NgF3′5′ H1‐4* exhibited low CHH methylation (13.41), while competing *NgFLSs* showed 9.76 and *NgDFRs* showed 13.80 (Figure 2H). This reciprocal methylation pattern between DHM pathway genes and their competing counterparts was consistently observed across all tissues examined.

Integrated methylome‐transcriptome analysis revealed a significant inverse correlation between promoter CHH methylation and gene expression (Spearman's *r* = –0.63, *p* < 0.01) (Figure S25). To explore the mechanistic basis, we examined the expression of DNA methylation machinery [14, 15] (Figure S26 and Table S54). Young leaves showed reduced transcript abundance of several CHH methylation‐related components: NgAGO4 (1.2‐fold lower than roots), NgDRM1 (1.2‐fold), NgCMT2 (1.4‐fold), NgCMT3 (2.9‐fold), and NgORTH2 (5.9‐fold), together with upregulation of the demethylase NgDML1 (1.3‐fold) (Figure S27 and Table S55). These correlative observations are consistent with the hypothesis that downregulation of the RdDM pathway contributes to reduced CHH methylation, which may permit high expression of duplicated DHM genes. However, direct causality remains to be tested (e.g., via targeted epigenome editing). Importantly, none of the previous genomic studies [5, 6] have explored this epigenetic layer, highlighting the unique contribution of our methylome resource.

## 3 A “DUPLICATION‐ASSOCIATED ACTIVATION” HYPOTHESIS

Integrating the genomic, epigenomic, and transcriptomic findings, we propose a “duplication‐associated activation” hypothesis for high DHM accumulation in vine tea. The hypothesis has three layers. First, at the genetic level, lineage‐specific tandem duplications have expanded the copy number of DHM pathway genes, especially *NgCHS* and *NgF3′5′ H*, providing increased catalytic capacity for DHM biosynthesis (Figure 1E,F). Second, at the epigenetic level, tissue‐specific CHH hypomethylation in young leaves correlates with the selective high expression of these duplicated genes. As detailed above, this hypomethylated state is associated with downregulation of RdDM‐related methyltransferases and upregulation of the demethylase NgDML1 (Figure S27), suggesting a mechanistic link. Third, at the transcriptional level, we identified candidate MYB and bHLH transcription factors [16, 17] (Figures 2I and S28, Tables S56–S58). Through differential expression analysis, Weighted Gene Co‐Expression Network Analysis (WGCNA), and Pearson correlation, three MYB candidates (NgMYB1, NgMYB2, NgMYB4) and one bHLH candidate (NgbHLH1) exhibited young leaf‐specific expression and strong co‐expression with DHM biosynthetic genes (Pearson's *r* > 0.8) (Figure 2J,K and S29, Tables S59–S62). Sequence homology analysis revealed that NgMYB1 shares 96.06% identity with VvMYB30, a known regulator of the competing stilbene pathway; NgMYB2 and NgMYB4 share 86.85%–89.15% identity with VvMYBA1, a known activator of the anthocyanin pathway; and NgbHLH1 shares 94.95% identity with VvbHLH71, which is implicated in flavonoid metabolism (Table S63) [18, 19]. These results suggest a potentially neofunctionalized role for these factors in activating DHM biosynthesis. However, while these correlative findings are consistent with our hypothesis, direct causality remains to be experimentally tested.

## 4 LIMITATIONS AND OUTLOOK

The genome and methylome resources presented here provide new insights into the regulatory mechanisms underlying high DHM accumulation in vine tea. Our findings suggest a multi‐layered framework in which tandem duplication, tissue‐specific CHH hypomethylation, and candidate transcription factors may collectively contribute to this trait.

The prevalence of tandem duplication among DHM pathway genes (96.4%) is striking, especially given the absence of whole‐family expansion across Vitaceae. This pattern aligns with the view that localized genomic rearrangements often drive metabolic specialization [8]. The functional retention of duplicated *NgCHS* and *NgF3′5′ H* copies supports that copy number expansion provides enhanced catalytic potential. However, tandem duplication alone cannot explain tissue‐specificity, as Vitis species possess similar duplications yet do not hyperaccumulate DHM, pointing to the importance of regulatory layers. Based on our methylome data, we speculate that the absence of comparable tissue‐specific CHH hypomethylation in Vitis may contribute to this difference, a hypothesis warranting future comparative epigenomic investigation. The correlation between CHH hypomethylation and high expression of DHM pathway genes, together with downregulation of RdDM‐related methyltransferases and upregulation of NgDML1, suggests an epigenetic contribution to tissue‐specific activation. Consistent with this view, DNA methylation is known to fine‐tune specialized metabolism in response to developmental cues [13], and our methylome resource adds an entirely new layer to existing genomic studies [5, 6].

Several opportunities for future investigation emerge from this work. Testing causality will require targeted epigenome editing (e.g., CRISPR‐dCas9 to demethylate NgCHS promoters), along with functional assays such as Y1H, ChIP‐qPCR, or stable transformation of candidate TFs. Small RNA profiling could identify 24‐nt siRNAs guiding RdDM to these loci. The large number of CHH DMRs, while reflecting the genome‐wide abundance of CHH sites, calls for focused analysis on genic and promoter regions where the biological signal is clearest, as we have done here.

In conclusion, this work provides a high‐quality genome and the first methylome for *N. grossedentata*, together with a testable “duplication‐associated activation” hypothesis. These resources should facilitate future mechanistic studies and metabolic engineering of DHM production.

## AUTHOR CONTRIBUTIONS

**Yingmei Wu**: Investigation; methodology; validation; visualization; software; formal analysis; writing—original draft; writing—review and editing. **Hanrui Hu**: Methodology; validation. **Qing Ye**: Investigation; software. **Xueqing Chen**: Investigation; software. **Miyuan Tian**: Resources. **Jing Li**: Conceptualization. **Li Lu**: Conceptualization. **Yifei Liu**: Conceptualization. **Yi Shang**: Conceptualization; supervision. **Yanling Li**: Conceptualization; supervision; writing—review and editing. **Xingyao Xiong**: Conceptualization; funding acquisition; supervision; project administration; writing—review and editing. **Junbo Gou**: Conceptualization; funding acquisition; supervision; software; formal analysis; project administration; writing—review and editing. All authors have read the final manuscript and approved it for publication.

## CONFLICT OF INTEREST STATEMENT

The authors declare no conflicts of interest.

## ETHICS STATEMENT

No animals or humans were involved in this study.

## Supporting information


**Figure S1:** Genome survey and Hi‐C interaction map of *Nekemias grossedentata*.
**Figure S2:** Comparative analysis of DHM biosynthetic gene families across Vitaceae species.
**Figure S3:** Chromosomal distribution of DHM biosynthetic genes in *Nekemias grossedentata*.
**Figure S4:** Modes of gene duplication contributing to the expansion of the DHM biosynthetic pathway.
**Figure S5:** Quantification of major flavonoids in different tissues of *Nekemias grossedentata* by HPLC.
**Figure S6:** Expression profiles of DHM biosynthetic genes and competing branch genes in young leaves.
**Figure S7:** Average DNA methylation levels across gene bodies and transposable elements (TEs) in root, stem, and young leaf tissues.
**Figure S8:** Genomic distribution of tissue‐specific DMRs across features like promoters, genes, and TEs.
**Figure S9:** Tissue‐specific DNA methylation levels in genomic functional regions.
**Figure S10:** KEGG pathway enrichment analysis of genes associated with hypomethylated DMRs in root and stem tissues of *Nekemias grossedentata*.
**Figure S11:** Schematic diagram of the DHM biosynthetic pathway in *Nekemias grossedentata* with key enzymes highlighted.
**Figure S12:** DNA methylation patterns of *Ng*CHS genes in *Nekemias grossedentata* tissues.
**Figure S13:** Methylation patterns and expression levels of key *NgF3′5′H* genes in the DHM biosynthetic pathway.
**Figure S14:** Methylation patterns and expression levels of key *NgF3H* genes in the DHM biosynthetic pathway.
**Figure S15:** Methylation patterns and expression levels of the key *NgF3′H1* gene in the DHM biosynthetic pathway.
**Figure S16:** Methylation patterns and expression levels of the key *NgCHI1* genes in the DHM biosynthetic pathway.
**Figure S17:** Hypermethylation of competing pathway *NgHCT* genes in young leaves of *Nekemias grossedentata*.
**Figure S18:** Sustained hypermethylation of *NgHCT* genes in the competing metabolic branch of *Nekemias grossedentata*.
**Figure S19:** DNA methylation patterns of *NgSTS* genes (*NgSTS1–NgSTS8*) in the competing metabolic branch of *Nekemias grossedentata*.
**Figure S20:** DNA methylation patterns of *NgSTS* genes (*NgSTS9–NgSTS16*) in the competing metabolic branch of *Nekemias grossedentata*.
**Figure S21:** DNA methylation patterns of *NgSTS* genes (*NgSTS17–NgSTS24*) in the competing metabolic branch of *Nekemias grossedentata*.
**Figure S22:** DNA methylation patterns of *NgSTS* genes (*NgSTS25–NgSTS28*) in the competing metabolic branch of *Nekemias grossedentata*.
**Figure S23:** Sustained promoter hypermethylation of downstream *NgFLS* genes in *Nekemias grossedentata*.
**Figure S24:** Sustained promoter hypermethylation of downstream *NgDFR* genes in *Nekemias grossedentata*.
**Figure S25:** Negative correlation between promoter methylation and gene expression for DHM biosynthetic genes in *Nekemias grossedentata*.
**Figure S26:** Expression patterns of DNA methyltransferases and demethylases in different tissues of *Nekemias grossedentata*.
**Figure S27:** Phylogenetic analysis and expression of DNA methyltransferases and demethylases in *Nekemias grossedentata*.
**Figure S28:** Transcription factor landscape associated with DHM biosynthesis in *Nekemias grossedentata*.
**Figure S29:** Gene dendrogram and module colors in blocks 1*–*6 of WGCNA.

**Table S1:** Summary of sequencing data used for genome assembly.
**Table S2:** K‐mer analysis statistics for genome size and heterozygosity estimation.
**Table S3:** Statistics of the PacBio HiFi assembly.
**Table S4:** The lengths of pseudochromosomes generated in the Hi‐C assembly.
**Table S5:** The Hi‐C assembly and annotation statistics of the vine tea genome.
**Table S6:** BUSCO analysis of genome assembly and annotation (busco_lineage: embryophyta_odb10).
**Table S7:** Statistics of Illumina mapping.
**Table S8:** Functional annotation of vine tea genes.
**Table S9:** Non‐coding RNA annotation statistics.
**Table S10:** Statistics of the number of gene families in *Nekemias grossedentata* and 15 other species.
**Table S11:** Classification of gene duplication events and expanded gene families.
**Table S12:** Key KEGG enrichment analysis of the expanded genes in *Nekemias grossedentata*.
**Table S13:** Gene families identified in the *Nekemias grossedentata* genome that encode key enzymes involved in phenylpropanoid‐flavonoid (PF) biosynthesis.
**Table S14:** Genes identified in the *Nekemias grossedentata* genome that encode key enzymes involved in phenylpropanoid‐flavonoid (PF) biosynthesis.
**Table S15:** Copy number of key enzyme‐encoding genes in the DHM biosynthetic pathway of *Nekemias grossedentata*.
**Table S16:** Comparative analysis of gene family sizes in the DHM pathway across three Vitaceae species (*Vitis vinifera*, *Vitis riparia*, and *Tetrastigma hemsleyanum*).
**Table S17:** Duplication modes of dihydromyricetin (DHM) biosynthetic genes.
**Table S18:** Pairwise protein sequence identity (%) among *NgCHS* paralogs.
**Table S19:** Divergence time estimates for the CHS gene duplication event in *N. grossedentata*.
**Table S20:** Pairwise protein sequence identity (%) among *NgF3′5′H* paralogs.
**Table S21:** Divergence time estimates for the *F3′5′H* gene duplication event in *Nekemias grossedentata*.
**Table S22:** Inventory and functional annotation of cytochrome P450 (CYP450) genes in *Nekemias grossedentata*.
**Table S23:** Inventory and functional annotation of 2‐oxoglutarate‐dependent dioxygenase (2‐ODD) genes in *Nekemias grossedentata*.
**Table S24:** Inventory of key enzymes in the dihydromyricetin biosynthetic pathway of *Nekemias grossedentata*.
**Table S25:** HPLC quantification of major flavonoids in vine tea tender leaves.
**Table S26:** Global DNA methylation levels in CG, CHG, and CHH sequence contexts across root, stem, and young leaf tissues.
**Table S27:** Genome‐wide distribution statistics of CG methylation levels across gene regions.
**Table S28:** Genome‐wide distribution statistics of CHG methylation levels across gene regions.
**Table S29:** Genome‐wide distribution statistics of CHH methylation levels across gene regions.
**Table S30:** Genome‐wide distribution statistics of CG methylation levels in repetitive sequences.
**Table S31:** Genome‐wide distribution statistics of CHG methylation levels in repetitive sequences.
**Table S32:** Genome‐wide distribution statistics of CHH methylation levels in repetitive sequences.
**Table S33:** The number of hyper‐ and hypo‐methylated differentially methylated regions (DMRs) in root, stem, and young leaf tissues.
**Table S34:** The number of hyper‐ and hypo‐DMRs overlapping with various genomic features (e.g., Exon, Intergenic, Intron, Promoter) in root, stem, and young leaf tissues.
**Table S35:** DNA methylation levels (CG, CHG, CHH) in exon, intron, intergenic, and promoter of root, stem, and young leaf tissues.
**Table S36:** KEGG pathway enrichment analysis of genes associated with CG‐hypoDMRs in young leaves.
**Table S37:** KEGG pathway enrichment analysis of genes associated with CHG‐hypoDMRs in young leaves.
**Table S38:** KEGG pathway enrichment analysis of genes associated with CHH‐hypoDMRs in young leaves.
**Table S39:** KEGG pathway enrichment analysis of genes associated with CG‐hypoDMRs in roots.
**Table S40:** KEGG pathway enrichment analysis of genes associated with CHG‐hypoDMRs in roots.
**Table S41:** KEGG pathway enrichment analysis of genes associated with CHH‐hypoDMRs in roots.
**Table S42:** KEGG pathway enrichment analysis of genes associated with CG‐hypoDMRs in stems.
**Table S43:** KEGG pathway enrichment analysis of genes associated with CHG‐hypoDMRs in stems.
**Table S44:** KEGG pathway enrichment analysis of genes associated with CHH‐hypoDMRs in stems.
**Table S45:** Average CG methylation levels of DHM biosynthetic genes in young leaves.
**Table S46:** Average CHG methylation levels of DHM biosynthetic genes in young leaves.
**Table S47:** Average CHH methylation levels of DHM biosynthetic genes in young leaves.
**Table S48:** Average CG methylation levels of DHM biosynthetic genes in roots.
**Table S49:** Average CHG methylation levels of DHM biosynthetic genes in roots.
**Table S50:** Average CHH methylation levels of DHM biosynthetic genes in roots.
**Table S51:** Average CG methylation levels of DHM biosynthetic genes in stems.
**Table S52:** Average CHG methylation levels of DHM biosynthetic genes in stems.
**Table S53:** Average CHH methylation levels of DHM biosynthetic genes in stems.
**Table S54:** Inventory of DNA methyltransferase and demethylase genes identified in the *Nekemias grossedentata* genome.
**Table S55:** Average expression levels of DNA methyltransferases and demethylases in roots, stems, and leaves.
**Table S56:** Predicted transcription factor binding sites in the 2‑kb promoter regions of 28 DHM biosynthetic genes in *Nekemias grossedentata*.
**Table S57:** Distribution of putative transcription factor families associated with the 28 DHM biosynthetic genes in *Nekemias grossedentata*.
**Table S58:** Genome‑wide identification and annotation of transcription factors in *Nekemias grossedentata*.
**Table S59:** KEGG pathway enrichment analysis of genes in the Red module.
**Table S60:** Pearson's correlation coefficients between candidate transcription factors and key dihydromyricetin biosynthetic genes.
**Table S61:** Significantly differentially expressed genes between root and young leaf tissues with functional annotations.
**Table S62:** Differentially expressed MYB and bHLH transcription factor candidates in young leaves versus roots.
**Table S63:** Shortlisted MYB and bHLH transcription factor candidates with NCBI BLAST annotations.
**Table S64:** Primer sequences for candidate *NgCHS* and *NgF3′5′ H* genes in *Nekemias grossedentata*.

## Data Availability

Raw sequencing data have been deposited in the Genome Sequence Archive (GSA) under BioProject PRJCA054666 (https://ngdc.cncb.ac.cn/bioproject/browse/PRJCA054666). All supporting data are available in the supplementary materials. The data and scripts used are saved in https://github.com/1YingmeiWu/Vine-tea-genome-methylome. Supplementary materials (methods, figures, tables, graphical abstract, slides, videos, Chinese translated version and update materials) may be found in the online DOI or iMeta Science http://www.imeta.science/imetaomics.
