# Supplementary material for: Chromosome‐level genome and methylome of vine tea suggest roles for tandem duplication and CHH hypomethylation in high dihydromyricetin accumulation
Source: IMetaOmics. 2026 Aug 6;3(3):e70129. doi: 10.1002/imo2.70129 (PMC13674461; doi:10.1002/imo2.70129)
Supplement: Supplementary file 1 [file IMO2-3-e70129-s002.docx]

Supporting information to

**Chromosome-level genome and methylome of vine tea suggest roles for tandem duplication and CHH hypomethylation in high dihydromyricetin accumulation**

**Running title:** Tandem duplication and CHH hypomethylation in vine tea

Yingmei Wu^1^, Hanrui Hu^1^, Qing Ye^1^, Xueqing Chen^2^, Miyuan Tian^3^, Jing Li^4^, Li Lu^5^, Yifei Liu^1^, Yi Shang^6^, Yanling Li^7*^, Xingyao Xiong^7,^ ^8*^, and Junbo Gou^1*^

^1^ Hubei Shizhen Laboratory, Hubei Key Laboratory of Resources and Chemistry of Chinese Medicine, College of Pharmacy, Hubei University of Chinese Medicine, Wuhan 430065, China

^2^ Shenzhen Huayi Xinchuang Biotechnology Co., Ltd., Shenzhen 518000, China

^3^ Laifeng County Rural Investment and Construction Co., Ltd., Enshi 445700, China

^4^ State Key Laboratory of Plant Diversity and Specialty Crops, Wuhan Botanical Garden, Chinese Academy of Sciences, Wuhan 430074, China

^5^ State Key Laboratory of Hybrid Rice, School of Pharmaceutical Sciences, Wuhan University, Wuhan 430071, China

^6^ Engineering Research Center for Valorization of Unique Bio-Resources in Yunnan, Ministry of Education, Yunnan Normal University, Kunming 650500, China

^7^ College of Horticulture, Hunan Agricultural University, Changsha 410128, China

^8^ Shenzhen Branch, Guangdong Laboratory for Lingnan Modern Agriculture, Shenzhen Key Laboratory of Agricultural Synthetic Biology, Agricultural Genomics Institute at Shenzhen, Chinese Academy of Agricultural Sciences, Shenzhen 518124, China

*Correspondence: liyanlin@hunau.edu.cn [(Yanling Li);](mailto:(liyanlin@hunau.edu.cn);) [xiongxingyao@caas.cn](mailto:xiongxingyao@caas.cn) (Xingyao Xiong); [junbogou@163.com](mailto:junbogou@163.com) ( Junbo Gou)

**METHODS**

## Plant materials and sample collection

Wild *Nekemias grossedentata* plants were collected from Guangxi, China (GPS: 24°32′21″N, 108°23′27″E) and maintained under controlled conditions at the Horticultural Conservation Center of Wuhan Botanical Garden, Chinese Academy of Sciences. For multi-omics analyses, tissues from five distinct organs, young leaves, mature leaves, stems, tendrils, and roots, were separately collected from three biological replicates. All samples were immediately flash-frozen in liquid nitrogen and stored at −80 ℃ until use.

## Genome sequencing, assembly, and annotation of N. grossedentata

High-molecular-weight genomic DNA was extracted from young leaves using a modified CTAB (Cetyltrimethylammonium Bromide) method. A multi-platform sequencing strategy was employed: (1) Illumina paired-end sequencing (NovaSeq 6000) generated 150-bp reads for genome survey and polishing; (2) PacBio HiFi sequencing (Sequel II system) produced ~15-kb circular consensus sequencing (CCS) for de novo assembly; (3) Hi-C libraries (Illumina NovaSeq) were constructed for chromosome scaffolding [1,2].

The genome size and heterozygosity were estimated using Illumina reads with GCE (Genome Characteristics Estimation) (v1.0.2). *De novo* assembly was performed from PacBio CCSs using Hifiasm (v0.16.1) with default parameters [3]. The primary assembly was polished using the Illumina reads with Pilon (v1.24) [4]. Hi-C reads were processed with Juicer (v1.6) and 3D-DNA (v180922) for scaffolding, contig ordering, and orientation, resulting in a chromosome-scale assembly [5,6]. Assembly quality was assessed by BUSCO (Benchmarking Universal Single-Copy Orthologs) (v5.3.2) against the embryophyta_odb10 database and by mapping Illumina reads back to the assembly using BWA-MEM (Burrows-Wheeler Aligner - Maximal Exact Matches) (v0.7.17) [7].

Repetitive elements were identified by a combination of homology-based searches against RepBase (v20181026) using RepeatMasker (v4.0.9) and *de novo* prediction with RepeatModeler (v1.0.11) [8,9]. Protein-coding genes were predicted using an evidence-driven pipeline: (1) Transcriptome-assisted prediction using RNA-seq data from multiple tissues assembled with Trinity (v2.14.0) and aligned with HISAT2 (Hierarchical Indexing for Spliced Alignment of Transcripts 2) (v2.1.0) [1,10]; (2) Homology-based prediction using protein sequences from *Vitis vinifera*, *Arabidopsis thaliana*, and *Oryza sativa* with GeMoMa (Gene Model Mapper) (v1.8) [11]; (3) Ab initio prediction using BRAKER2 (v2.1.6). All predictions were integrated with EVidenceModeler (v1.1.1) [12,13]. Gene functions were annotated by aligning protein sequences against the NR, Swiss-Prot, and KEGG databases using BLASTP (v2.12.0), and InterProScan (v5.52-86.0) was used for protein domain identification [14-18]. To systematically identify components of the DNA methylation machinery, DNA methyltransferases and demethylases were annotated by searching the vine tea proteome against reference sequences from *Arabidopsis thaliana*, *Vitis vinifera*, and other plant species using a combination of BLASTP (Basic Local Alignment Search Tool for Proteins) and conserved domain analysis (Pfam) [19]. Transcription factors (TFs) were identified by screening against the Plant Transcription Factor Database (PlantTFDB) using hmmscan (HMMER v3.3.2) with default cutoffs [20,21]. The expression patterns of these identified genes across tissues were further characterized using RNA-seq data.

## Comparative genomics and synteny analysis

Comparative synteny analysis was performed to investigate the genomic context surrounding the target genes in Amega, Vvini, and Vripa. Pairwise homologous gene relationships and conserved syntenic blocks were identified using the JCVI toolkit (version 1.5.4), a Python-based toolkit for comparative genomics analysis. The resulting syntenic blocks were filtered to retain regions containing the target genes. Gene positional information was provided in BED format, and the arrangement of the compared genomic regions was specified in a layout file. The filtered syntenic blocks were visualized using the jcvi.graphics.synteny module with the following command:python3 -m jcvi.graphics.synteny \Ameg.blocks.txt \ gene.bed \ layout.csv \ --genelabelsize=6 \ --genelabels="TARGET_GENE_1, TARGET_GENE_2, TARGET_GENE_3". Here, Ameg.blocks.txt contains the filtered conserved syntenic blocks, gene.bed contains the genomic coordinates and gene names, and layout.csv defines the layout of the synteny plot. The target gene names in the command were replaced by the genes displayed in Figure 1E. The synteny analysis and visualization were performed according to the JCVI toolkit documentation and the corresponding software publication [22].

## RNA sequencing and expression analysis in N. grossedentata

Total RNA from root, stem, and young leaf tissues (three biological replicates each) was extracted using the MiniBEST Plant RNA Extraction Kit (Takara). RNA quality was assessed (RIN > 7.0 for all samples), and sequencing libraries were prepared and sequenced on the DNBSEQ platform (BGI). Clean reads were aligned to the *N. grossedentata* reference genome using HISAT2 (v2.2.1). Read counts per gene were obtained using featureCounts (v2.0.1) [23].

Differential gene expression analysis between tissues was performed with DESeq2 (v1.26.0) using the design formula ~ Tissue, where Tissue is a factor with three levels (root, stem, young leaf) [24]. The normalization procedure was as follows: (1) log-transformation of read counts (0 values remained 0); (2) calculation of mean expression for each gene across all samples; (3) subtraction of the mean from each gene's expression per sample; (4) median calculation per sample; (5) exponential transformation of medians to obtain size factors; (6) division of raw read counts by size factors to obtain normalized expression values. Differentially expressed genes (DEGs) were identified using the Wald test with a negative binomial model for dispersion estimation, and *p*-values were adjusted using the Benjamini-Hochberg procedure. Genes with |log_2_FC| > 1 and adjusted *p*-value (*p*adj) < 0.05 were considered significantly differentially expressed.

For quality control, principal component analysis (PCA), sample-to-sample Pearson correlation analysis, and boxplots of normalized counts were performed to evaluate sample clustering, reproducibility, and data dispersion. No outliers were detected, and no samples were excluded from the analysis. For visualization in figures, normalized expression values are presented as fragments per kilobase of transcript per million mapped reads (FPKM).

Weighted gene co-expression network analysis (WGCNA) was conducted using the WGCNA R package (v1.72) to identify modules of co-expressed genes [25]. Briefly, a signed adjacency matrix was constructed using a soft-thresholding power of 12 (scale-free topology fit index R^2^ = 0.89), and the topological overlap matrix (TOM) was used for hierarchical clustering. Modules were defined with a minimum module size of 30 and a cut height of 0.25. Module-trait associations were calculated by correlating module eigengenes with tissue types (root, stem, young leaf). Hub transcription factors within modules of interest were identified based on high module membership (MM > 0.8) and gene significance (GS > 0.8).

## Whole-genome bisulfite sequencing (WGBS) and DNA methylation analysis

**Whole-genome bisulfite sequencing (WGBS) and DNA methylation analysis were performed as previously described [26,27]. Genomic DNA from root, stem, and young leaf tissues (*n* = 3) was extracted, and bisulfite conversion was performed using the EZ DNA Methylation-Gold Kit (Zymo Research). WGBS libraries were constructed with the Accel-NGS Methyl-Seq DNA Library Kit (Swift Biosciences) and sequenced on the Illumina NovaSeq 6000 platform (150-bp paired-end reads).**

**Raw reads were filtered and trimmed for adapters and low-quality bases using fastp, yielding clean reads for downstream analysis. Bisulfite conversion efficiency was assessed by spiking in 1% unmethylated lambda DNA, resulting in a conversion rate > 99.5% for all samples. Clean reads were aligned to the *N. grossedentata* reference genome using Bismark (v0.24.0) with bowtie2 [28,29]. Methylation levels for individual cytosines in CG, CHG, and CHH contexts were extracted using the bismark_methylation_extractor tool, requiring a minimum coverage of 10 reads per site. Coverage distribution across samples was examined, with a median sequencing depth of ≥ 30× per sample. Sample-to-sample Pearson correlation and principal component analysis (PCA) based on methylation levels across all sites confirmed high reproducibility among biological replicates. Differentially methylated regions (DMRs) were identified using the DSS R package with the following criteria: |Δmethylation| > 0.2 and false discovery rate (FDR) < 0.05, controlled using the Benjamini-Hochberg procedure [30]. DSS was run with default window and smoothing parameters. DMR-associated genes were subjected to KEGG enrichment analysis using clusterProfiler [31]. The input gene list comprised genes overlapping with DMRs (gene body ± 2 kb promoter region). The background gene set was all protein-coding genes with KEGG annotations in the *N. grossedentata* genome. To integrate DNA methylation with gene expression, a comprehensive methylome-transcriptome analysis was performed by overlapping DMRs (especially in promoter regions) with differentially expressed genes (DEGs). The correlation between methylation levels and gene expression was assessed using scatter plots and Pearson correlation coefficients.**

## Quantification of flavonoids by high-performance liquid chromatography (HPLC)

Fresh tissues (root, stem, young leaf; ~1.0 g fresh weight each) were ground in liquid nitrogen and extracted with 8 mL of 80% methanol (v/v) by sonication for 45 min. The extract was centrifuged, filtered through a 0.22-μm PTFE membrane, and concentrated under vacuum. HPLC analysis was performed on an Agilent 1260 Infinity II system equipped with a TC-C18 column (250 × 4.6 mm, 5 μm; Agilent). The mobile phase consisted of (A) 0.1% formic acid in water and (B) acetonitrile. The gradient elution program was: 0–5 min, 20–40% B; 5–10 min, 40–50% B; 10–20 min, 50–80% B; 20–25 min, 80% B; 25–30 min, return to 20% B. The flow rate was 1.0 mL·min^–1^, column temperature was 30 °C, and detection wavelength was 290 nm. Quantification was based on external calibration curves of authentic standards (DHM, dihydromyricetin; DHQ, dihydroquercetin; MYR, myricetin; QUE, quercetin). Three biological replicates were analyzed, each with three technical replicates.

## Cloning and transient expression in Nicotiana benthamiana

**The coding sequences of *NgCHS1, NgCHS2, NgCHS3, NgF3′5′H1, NgF3′5′H3*, and *NgF3′5′H4* were amplified from vine tea cDNA. The *LjCPR* (cytochrome P450 reductase) gene from *Lotus japonicus* (AB433810) was synthesized. All genes were cloned into the plant expression vector PEAQ-HT via the AgeI and XhoI sites. Recombinant plasmids were verified by Sanger sequencing and transformed into Agrobacterium tumefaciens strain GV3101. Primer sequences are provided in Table S64.**

## **Agrobacterium tumefaciens strain GV3101 harboring the respective constructs was grown to an OD_600_ of 0.6–0.8, harvested, and resuspended in infiltration buffer (10 mM MES, pH 5.6, 10 mM MgCl_2_, 100 µM acetosyringone). After incubation at room temperature for 2–3 h, the bacterial suspensions were infiltrated into the abaxial side of leaves from 4–5-week-old N. benthamiana plants using a needleless syringe [32,33].**

## **For all functional assays, the same two-step timeline was used: (i) agroinfiltration of N. benthamiana leaves with the respective constructs; (ii) 4 days post-agroinfiltration, the corresponding substrates were infiltrated into the same leaf areas; (iii) 48 hours after substrate infiltration, leaf samples were harvested for analysis. Specifically, for NgCHS functional assays, a mixture of malonyl-CoA (50 µg·mL^–1^) and p-coumaroyl-CoA (50 µg·mL^–1^) was infiltrated. For NgF3′5′H assays, dihydroquercetin (100 µM) was infiltrated into leaves co-expressing NgF3′5′Hs and LjCPR. Three independent infiltration experiments were performed for each construct.**

## Metabolite analysis by UHPLC-Q-TOF-MS

Infiltrated *N. benthamiana* leaf tissues (0.5 g) were extracted with 10 mL methanol by ultrasonication for 1 h. The extract was centrifuged (**12,000 × *g,* 10 min)**, filtered, and analyzed on an ACQUITY UPLC M-Class system coupled to a Xevo G2-XS Q-ToF mass spectrometer (Waters). Separation was performed on an ACQUITY UPLC BEH C18 column (1.7 μm, 2.1 × 100 mm) with a gradient of 0.1% formic acid in water (A) and 0.1% formic acid in acetonitrile (B) at a flow rate of 0.5 mL·min^–1^ **and the injection volume was 2 µL**. **The elution gradient was as follows: 5% B (0–0.5 min), 5% to 98% B (0.5–11 min), 100% B (11–13 min), 5% B (13–17 min).** Mass spectrometry data were acquired in positive electrospray ionization mode. Metabolites were identified by comparison of retention times and mass spectra with authentic standards and quantified using extracted ion chromatograms. Three biological replicates were analyzed.

## Data Analysis

All experiments were performed with at least three independent biological replicates, as indicated in the respective figure legends. Data are presented as mean ± standard deviation (SD). Normality of continuous data was assessed using the Shapiro-Wilk test (α = 0.05). Homogeneity of variances among groups was evaluated using Levene’s test (α = 0.05). All statistical tests were two-sided unless otherwise specified.

For Figure 1H,J (quantification of naringenin chalcone and DHM production in *N. benthamiana*): One-way ANOVA was used to compare metabolite levels among multiple expression constructs (NgCHS1/2/3 for Figure 1H; NgF3′5′H1/3/4 for Figure 1J) and the empty vector control. Tukey’s honestly significant difference (HSD) post-hoc test was applied for pairwise comparisons against the control group. Normality and variance homogeneity were confirmed prior to ANOVA. Three independent infiltration experiments were analyzed (*n* = 3 biological replicates per construct).

For Figure 2D (KEGG enrichment of genes associated with CHH-hypo-DMRs in young leaves): Hypergeometric test was implemented using the enrich KEGG function in clusterProfiler (v4.4.0). The input gene list comprised genes overlapping with CHH-hypo-DMRs in young leaves (gene body ± 2 kb promoter). The background was all protein-coding genes with KEGG orthology annotations in the *N. grossedentata* genome. The comparison of interest was young leaves versus roots (*i.e.*, genes hypomethylated in young leaves relative to roots). Multiple testing correction was performed using the Benjamini-Hochberg procedure, and adjusted *p*-values (FDR, *p* < 0.05) were considered statistically significant. All “significant enrichment” reported in the main text and figures are based on adjusted *p*-values (*p*-value) rather than raw *p*-values.

For Figure S7 (average methylation levels across gene bodies and TEs): One-way ANOVA was used to compare methylation levels among the three tissues (root, stem, young leaf) separately for each sequence context (CG, CHG, CHH) and each genomic feature (gene body, TE). Normality was verified by Shapiro-Wilk test, and homogeneity of variances by Levene’s test. Tukey’s HSD post-hoc test was applied for pairwise comparisons. Statistical significance was set at α = 0.05.

For Figure S10 (KEGG enrichment of DMR-associated genes in root and stem tissues): Hypergeometric test followed by Benjamini-Hochberg FDR correction (*p* < 0.05) was performed using enrichKEGG in clusterProfiler. The input gene lists varied by tissue (root, stem) based on tissue-specific DMR-associated genes (gene body ± 2 kb promoter). The background was all protein-coding genes with KEGG orthology annotations in the *N. grossedentata* genome. The comparison for root DMRs was roots versus young leaves; for stem DMRs, stems versus young leaves. All reported enrichment significance is based on adjusted *p*-values (*p*-value).

For Figures S12–S24 (methylation profiles of individual gene families): For each gene and each sequence context (CG, CHG, CHH), average methylation levels were compared among root, stem, and young leaf using one-way ANOVA with Tukey’s HSD post-hoc test, after confirming normality and variance homogeneity as described above. For promoter-specific comparisons, methylation levels were averaged over the 2-kb region upstream of the transcription start site.

For Figure S25 (correlation between promoter methylation and gene expression): Spearman’s rank correlation coefficient (*r*) was used because the combined dataset of 28 DHM pathway genes did not meet bivariate normality assumptions (Shapiro-Wilk test, *p* < 0.05 for at least one variable). A two-tailed test was performed using the cor.test function in r (method = “spearman”). No multiple testing correction was applied for this targeted gene set. The 95% confidence interval for ρ was calculated using Fisher’s Z-transformation.

For Figures S26 and S27 (expression of methyltransferases and demethylases): Pairwise comparisons between young leaf and other tissues for each gene were performed using unpaired two-tailed *t*-tests after confirming normality (Shapiro-Wilk) and homogeneity of variances (Levene’s test). When variances were unequal, Welch’s *t*-test was used. Benjamini-Hochberg correction was applied for multiple comparisons across genes within each figure (FDR < 0.05).

For Figure S29 (WGCNA module detection): No formal statistical testing was applied to module assignment. Scale-free topology fit (R^2^) and mean connectivity were used as descriptive diagnostics. Module-trait associations were assessed by Pearson correlation between module eigengene and tissue type; *p*-values were adjusted using Benjamini-Hochberg correction within modules.

For HPLC quantification (Figure S5 and associated supplementary tables): Three biological replicates were analyzed, each with three technical replicates. Technical replicates were averaged per biological replicate before statistical analysis. Comparison of flavonoid contents (DHM, DHQ, MYR, QUE) across tissues was performed using one-way ANOVA with Tukey’s HSD post-hoc test, following normality and variance homogeneity checks.

For transient expression assays in *N. benthamiana*: Comparison of metabolite levels between empty vector control and each construct was performed using unpaired two-tailed Student’s *t*-test (assuming equal variances after Levene’s test). Three independent infiltration experiments were analyzed.

General notes: For RNA-seq differential expression analysis (Table S5), the Wald test implemented in DESeq2 (v1.26.0) was used with the design formula ~ Tissue. Regularized-logarithm transformation (rlog) was applied for visualization. Dispersion estimates were fitted to a trend line, and Cook’s distance was used to identify influential counts (default cutoff). Benjamini-Hochberg correction was applied to obtain adjusted *p*-values (*p.*adj). For all omics analyses, the unit of analysis was the biological replicate, with *n* = 3 per tissue unless otherwise stated.

**REFERENCES**

1. Grabherr, Manfred G, Brian J Haas, Moran Yassour, Joshua Z Levin, Dawn A Thompson, Ido Amit, Xian Adiconis, et al. 2011. “Full-length transcriptome assembly from RNA-Seq data without a reference genome.” *Nature Biotechnology* 29:644–652. [https://doi.org/10.1038/nbt.1883](https://doi.org/10.1038/nbt.1883.)
2. Zhang, Tianyuan, Hanzhou Li, Mian Jiang, Huiyu Hou, Yunyun Gao, Yali Li, Fuhao Wang, et al. 2024. “Nanopore sequencing: flourishing in its teenage years.” *Journal of Genetics and Genomics* 51(12): 1361–1374. <https://doi.org/10.1016/j.jgg.2024.09.0073>
3. Cheng, Haoyu, Gregory T Concepcion, Xiaowen Feng, Haowen Zhang, Heng Li. 2021. “Haplotype-resolved de novo assembly using phased assembly graphs with hifiasm.” *Nature Methods* 18(2): 170–175. <https://doi.org/10.1038/s41592-020-01056-5>
4. Walker, Bruce J, Thomas Abeel, Terrance Shea, Margaret Priest, Amr Abouelliel, Sharadha Sakthikumar, et al. 2014. “Pilon: an integrated tool for comprehensive microbial variant detection and genome assembly improvement.” *PloS One* 9(11): e112963. <https://doi.org/10.1371/journal.pone.0112963>
5. Durand, Neva C, Muhammad S Shamim, Ido Machol, Suhas S P Rao, Miriam H Huntley, Eric S Lander, et al. 2016. “Juicer provides a one-click system for analyzing loop-resolution Hi-C experiments.” *Cell Systems* 3(1): 95–98. <https://doi.org/10.1016/j.cels.2016.07.002>
6. Dudchenko, Olga, Sanjit S Batra, Arina D Omer, Sarah K Nyquist, Marie Hoeger, Neva C Durand, et al. 2017. “De novo assembly of the *Aedes aegypti* genome using Hi-C yields chromosome-length scaffolds.” *Science* 356(6333): 92–95. <https://doi.org/10.1126/science.aal3327>
7. Manni, Mosè, Matthew R Berkeley, Mathieu Seppey, Felipe A Simão, Evgeny M Zdobnov. 2021. “BUSCO update: novel and streamlined workflows along with broader and deeper phylogenetic coverage for scoring of Eukaryotic, prokaryotic, and viral genomes.” *Molecular Biology and Evolution* 38(10): 4647–4654. <https://doi.org/10.1093/molbev/msab199>
8. Weidong Bao, Kenji K Kojima, Oleksiy Kohany. 2015. “Repbase Update, a database of repetitive elements in eukaryotic genomes.” *Mobile DNA* 6: 11. <https://doi.org/10.1186/s13100-015-0041-9>
9. Flynn, Jullien M, Robert Hubley, Clément Goubert, Jeb Rosen, Andrew G Clark, Cédric Feschotte, et al. 2020. “RepeatModeler2 for automated genomic discovery of transposable element families.” *Proceedings of the National Academy of Sciences of the United States of America* 117(17): 9451–9457. <https://doi.org/10.1073/pnas.1921046117>
10. Kim, Daehwan, Joseph M Paggi, Chanhee Park, Christopher Bennett, Steven L Salzberg. 2019. “Graph-based genome alignment and genotyping with HISAT2 and HISAT-genotype.” *Nature Biotechnology* 37(8): 907–915. <https://doi.org/10.1038/s41587-019-0201-4.>
11. Keilwagen, Jens, Michael Wenk, Jessica L Erickson, Martin H Schattat, Jan Grau, Frank Hartung. 2016. “Using intron position conservation for homology-based gene prediction.” *Nucleic Acids Research* 44(9): e89. <https://doi.org/10.1093/nar/gkw092>
12. Tomáš Brůna, Katharina J Hoff, Alexandre Lomsadze, Mario Stanke, Mark Borodovsky. 2021. “BRAKER2: automatic eukaryotic genome annotation with GeneMark-EP+ and AUGUSTUS supported by a protein database.” *NAR Genomics and Bioinformatics* 3(1): lqaa108. https://doi.org/10.1093/nargab/lqaa108
13. Haas, Brian J, Steven L Salzberg, Wei Zhu, Mihaela Pertea, Jonathan E Allen, Joshua Orvis, et al. 2008. “Automated eukaryotic gene structure annotation using EVidenceModeler and the Program to Assemble Spliced Alignments.” *Genome Biology* 9(1): R7. <https://doi.org/10.1186/gb-2008-9-1-r7>
14. Camacho, Christiam, George Coulouris, Vahram Avagyan, Ning Ma, Jason Papadopoulos, et al. 2009. “BLAST+: architecture and applications.” *BMC Bioinformatics* 10: 421. <https://doi.org/10.1186/1471-2105-10-421.>
15. Sayers, Eric W, Evan E Bolton, J Rodney Brister, Kathi Canese, Jessica Chan, et al. 2022. “Database resources of the national center for biotechnology information.” *Nucleic Acids Research* 50(D1): D20–D26. <https://doi.org/10.1093/nar/gkab1112>
16. UniProt Consortium. 2023. “UniProt: the Universal Protein Knowledgebase in 2023.” *Nucleic Acids Research* 51(D1): D523–D531. <https://doi.org/10.1093/nar/gkac1052>
17. Kanehisa, Minoru, Miho Furumichi, Yoko Sato, Masayuki Kawashima, Mari Ishiguro-Watanabe. 2023. “KEGG for taxonomy-based analysis of pathways and genomes.” *Nucleic Acids Research* 51(D1): D587–D592. <https://doi.org/10.1093/nar/gkac963>
18. Jones, Philip, David Binns, Hsin-Yu Chang, Matthew Fraser, Weizhong Li, et al. 2014. “InterProScan 5: genome-scale protein function classification.” *Bioinformatics* 30(9): 1236–1240. <https://doi.org/10.1093/bioinformatics/btu031>
19. [Mistry](https://pubmed.ncbi.nlm.nih.gov/?term=Mistry+J&cauthor_id=33125078), Jaina, [Sara Chuguransky](https://pubmed.ncbi.nlm.nih.gov/?term=Chuguransky+S&cauthor_id=33125078), [Lowri Williams](https://pubmed.ncbi.nlm.nih.gov/?term=Williams+L&cauthor_id=33125078), [Matloob Qureshi](https://pubmed.ncbi.nlm.nih.gov/?term=Qureshi+M&cauthor_id=33125078), [Gustavo A Salazar](https://pubmed.ncbi.nlm.nih.gov/?term=Salazar+GA&cauthor_id=33125078), et al. 2021. “Pfam: The protein families database in 2021.” *Nucleic Acids Research* 49(D1): D412–D419. <https://doi.org/10.1093/nar/gkaa913>
20. Tian, Feng, De-Chang Yang, Yu-Qi Meng, Jinpu Jin, Ge Gao. 2020. “PlantRegMap: charting functional regulatory maps in plants.” *Nucleic Acids Research* 48(D1): D1104–D1113. <https://doi.org/10.1093/nar/gkz1020>
21. Eddy, Sean R. 2011. “Accelerated Profile HMM Searches.” *PLoS Computational Biology* 7(10): e1002195. [https://doi.org/10.1371/journal.pcbi.1002195](https://doi.org/10.1371/journal.pcbi.1002195.)
22. Tang, Haibao, Vivek Krishnakumar, Xiaofei Zeng, Zhougeng Xu, Adam Taranto, Johnathan S Lomas, Yixing Zhang, et al. 2024. “JCVI: A versatile toolkit for comparative genomics analysis.” *iMeta* 3(4): e211. <https://doi.org/10.1002/imt2.211>
23. Liao, Yang, Gordon K Smyth, Wei Shi. 2014. “featureCounts: an efficient general purpose program for assigning sequence reads to genomic features.” *Bioinformatics* 30(7): 923–930. <https://doi.org/10.1093/bioinformatics/btt656>
24. Love, Michael I, Wolfgang Huber, Simon Anders. 2014. “Moderated estimation of fold change and dispersion for RNA-seq data with DESeq2.” *Genome Biology* 15(12): 550. <https://doi.org/10.1186/s13059-014-0550-8>
25. Langfelder, Peter, Steve Horvath. 2008. “WGCNA: an R package for weighted correlation network analysis.” *BMC Bioinformatics* 9: 559. <https://doi.org/10.1186/1471-2105-9-559>
26. Zhang, Tianyuan, Jia Li, Chao Tang, You Wu, Hao Wu, Xi‐Tong Zhu, Ziyang Luo, et al. 2026. “Nanopore direct RNA sequencing and the epitranscriptome: advances in mapping native RNA landscapes.” *iMeta* e70136. https://doi.org/10.1002/imt2.70136
27. Gong, Ting, Heather Borgard, Zao Zhang, Shaoqiu Chen, Zitong Gao, Youping Deng. 2022. “Analysis and performance assessment of the whole genome bisulfite sequencing data workflow: currently available tools and a practical guide to advance DNA methylation studies.” *Small Methods* 6(3): e2101251. [https://doi.org/10.1002/smtd.202101251](https://doi.org/10.1002/smtd.202101251.)
28. Krueger, Felix, Simon R Andrews.2011. “Bismark: a flexible aligner and methylation caller for Bisulfite-Seq applications.” *Bioinformatics* 27(11): 1571–1572. <https://doi.org/10.1093/bioinformatics/btr167>
29. Langmead, Ben, Steven L Salzberg. 2012. “Fast gapped-read alignment with Bowtie 2.” *Nature Methods* 9(4): 357–359. <https://doi.org/10.1038/nmeth.1923>
30. Feng, Hao, Karen N Conneely, Hao Wu. 2014. “A Bayesian hierarchical model to detect differentially methylated loci from single nucleotide resolution sequencing data.” *Nucleic Acids Research* 42(8): e69. <https://doi.org/10.1093/nar/gku154>
31. Wu, Tianzhi, Erqiang Hu, Shuangbin Xu, Meijun Chen, Pingfan Guo, Zehan Dai, et al. 2021. “clusterProfiler 4.0: A universal enrichment tool for interpreting omics data.” *Innovation* 2(3): 100141. <https://doi.org/10.1016/j.xinn.2021.100141>
32. Wu, Yingmei, Xueqing Chen, Fuhua Hao, Yu Liu, Wei Luo, Yaru Zhu, Li Li, et al. 2024. “Biosynthesis of bridged tricyclic sesquiterpenes in *Inula lineariifolia*.” *The Plant Journal* 120(2): 658–673. <https://doi.org/10.1111/tpj.17008>
33. Zhang, Zhong, Yingmei Wu, Jiangyue Long, Qin Shi, Wei Tan, Hanrui Hu, Lingling Wei, et al. 2026. “Multi-omics dissection of the structural and biosynthetic diversity of steroidal glycoalkaloids in wild potatoes.” *Plant Physiology* 200(3): kiaf652. <https://doi.org/10.1093/plphys/kiaf652>

**Figure S1 Genome survey and Hi-C interaction map of *N. grossedentata*.** (A) *K*-mer frequency distribution (*k* = 19) of Illumina sequencing data. The major peak at 92.79 × coverage corresponds to an estimated genome size of 627.93 Mb. The heterozygosity rate is 0.85%, and the repeat content is 50.86%. (B) Hi-C contact map of the chromosome-scale genome assembly. The heatmap illustrates interaction frequencies between the 20 pseudochromosomes, with strong signals along the diagonal indicating accurate chromatin organization and high assembly quality. The color bar represents log-transformed interaction frequencies.

**Figure S2 Comparative analysis of DHM biosynthetic gene families across Vitaceae species**. Bar plot showing the copy number of key enzyme-encoding genes in the dihydromyricetin (DHM) pathway in *N. grossedentata* (Ng) compared to *T. Hemsleyanum* (Th)*, V. riparia* (Vr) and *V. vinifera* (Vv). Gene families include *PAL*, *C4H, 4CL,C3H,COMT, HCT, CAD, CHS, STS, CHI, F3H, FLS, F3'5'H, F3'H, DFR, ANR,* and *LAR*.

**Figure S3 Chromosomal distribution of DHM biosynthetic genes in *N. grossedentata*.** Genomic locations of key genes (*e.g.*, *PAL*, *C4H*, *4CL*, *CHS*, *CHI*, *F3'5'H*, *F3'H*, *F3H*, and *FLS*) involved in DHM biosynthesis across the 20 pseudochromosomes.

**Figure S4 Modes of gene duplication contributing to the expansion of the DHM biosynthetic pathway.** Pie chart summarizing the proportions of gene duplication events (WGD, whole‑genome duplication; TD, tandem duplication; PD, proximal duplication; TRD, transposed duplication; DSD, dispersed duplication). ‘Co’ indicates genes assigned to multiple duplication modes; ‘Uniq’ indicates genes uniquely assigned to a single mode.

**Figure S5 Quantification of major flavonoids in different tissues of *N. grossedentata* by HPLC.** Representative HPLC chromatograms of methanol extracts from roots, stems, and young leaves of *N. grossedentata*. Separation was performed on a TC-C18 column (250 × 4.6 mm, 5 μm) with detection at 290 nm. Peaks corresponding to dihydromyricetin (DHM), dihydroquercetin (DHQ), myricetin (MYR), and quercetin (QUE) are labeled. Quantification of peak areas from three biological replicates per tissue is provided in Table S25. DHM is the dominant flavonoid in young leaves, while its levels in roots and stems are substantially lower, demonstrating tissue-specific accumulation.

**Figure S6** **Expression profiles of DHM biosynthetic genes and competing branch genes in young leaves.** (A)Expression (FPKM) of *NgCHS1-3* versus competing branch genes (*NgHCTs*, *NgC3Hs*, *NgSTSs*). Combined expression of the three *NgCHS* genes exceeds that of the *NgSTS* gene family by > 570-fold. (B) Expression of *NgF3′5′H1-4, NgF3′H, NgF3Hs* versus competing downstream genes (*NgFLSs*, *NgDFRs*). *NgF3′5′H3* shows the highest expression (FPKM = 1020.76). Data are mean ± SD (*n* = 3). FPKM, fragments per kilobase per million.

**Figure S7 Average DNA methylation levels across gene bodies and transposable elements (TEs) in root, stem, and young leaf tissues.** (A) Average methylation levels across gene bodies (with 2 kb upstream and downstream flanking regions) in CG, CHG, and CHH contexts. Dashed lines indicate transcription start sites (TSS) and transcription end sites (TES). Methylation levels reach a minimum near TSS and gradually increase toward TES. (B) Average methylation levels across TEs (with 2 kb flanking regions) in CG, CHG, and CHH contexts. TE regions show elevated methylation levels compared to flanking regions, particularly in CHG and CHH contexts. Across both gene bodies and TEs, CHH methylation is consistently lower in young leaves than in roots and stems.

**Figure S8 Genomic distribution of tissue-specific DMRs across features like promoters, genes, and TEs.** Bar charts showing the distribution of hypo-DMRs across genomic features: intergenic regions, promoters, introns, and exons. Hypo-DMRs are predominantly enriched in intergenic regions, with a substantial proportion also located in promoters. Data are presented for young leaves, roots, and stems.

**Figure S9 Tissue-specific DNA methylation levels in genomic functional regions.** Average methylation levels in CG, CHG, and CHH sequence contexts in exons, introns, intergenic regions, and promoters of root, stem, and young leaf tissues.

**Figure S10 KEGG pathway enrichment analysis of genes associated with hypomethylated DMRs in root and stem tissues of *N. grossedentata*.** Bubble plot showing significantly enriched KEGG pathways (Benjamini-Hochberg adjusted *p*-value, *p* < 0.05) for genes associated with hypo-DMRs in leaf, root and stem tissues. Key metabolic pathways, including “flavonoid biosynthesis” and “phenylpropanoid biosynthesis”, are highlighted.

**Figure S11** **Schematic diagram of the DHM biosynthetic pathway in *N. grossedentata* with key enzymes highlighted.** The diagram illustrates the core flavonoid biosynthetic pathway leading to dihydromyricetin (DHM) accumulation. Key enzymes are shown in colored boxes: PAL (phenylalanine ammonia-lyase), C4H (cinnamate 4-hydroxylase), 4CL (4-coumarate-CoA ligase), CHS (chalcone synthase), CHI (chalcone isomerase), F3H (flavanone 3-hydroxylase), and F3′5′H (flavonoid 3′,5′-hydroxylase). Competing branch pathways (*e.g.*, stilbene pathway via STS, lignin pathway via HCT, and anthocyanin pathway via DFR) are indicated with dashed arrows. Enzymes that are tandem-duplicated in the vine tea genome (NgCHS and NgF3′5′H) are highlighted in red. The chemical structures of major intermediates and the final product DHM are shown below the pathway.

**Figure S12 DNA methylation patterns of *Ng*CHS genes in *N. grossedentata* tissues.** (A) Base-resolution methylation profiles (IGV snapshots) of *NgCHS*1, *NgCHS2*, and *NgCHS3* across root, stem, and young leaf tissues. Each row represents an individual CpG site, with methylation status color-coded as indicated. The purple bars represent gene structures, and the white arrows indicate the transcriptional direction of the genes. The displayed region encompasses the entire gene locus, from the transcription start site to the transcription termination site. Purple rectangles of different widths represent distinct gene structure regions: wider rectangles indicate exons, whereas narrower regions represent introns or untranslated regions (UTRs). (B) Average DNA methylation levels of the *NgCHS* gene family in CG, CHG, and CHH contexts across the three tissues. Data are presented as mean; *n* = 3 biological replicates.

**Figure S13 Methylation patterns and expression levels of key *NgF3’5’H* genes in the DHM biosynthetic pathway.** (A) Base-resolution methylation profiles (IGV snapshots) of *NgF3'5'H1–NgF3'5'H4* across root, stem, and young leaf tissues. The purple bars represent gene structures, and the white arrows indicate the transcriptional direction of the genes. The displayed region encompasses the entire gene locus, from the transcription start site to the transcription termination site. Purple rectangles of different widths represent distinct gene structure regions: wider rectangles indicate exons, whereas narrower regions represent introns or untranslated regions (UTRs). (B) Average DNA methylation levels in promoter regions of these *NgF3'5'H* genes across CG, CHG, and CHH contexts. Data are presented as mean; *n* = 3 biological replicates.

**Figure S14 Methylation patterns and expression levels of key *NgF3H* genes in the DHM biosynthetic pathway.** (A) Base-resolution methylation profiles (IGV snapshots) of *NgF3H1* and *NgF3H2* across root, stem, and young leaf tissues. The purple bars represent gene structures, and the white arrows indicate the transcriptional direction of the genes. The displayed region encompasses the entire gene locus, from the transcription start site to the transcription termination site. Purple rectangles of different widths represent distinct gene structure regions: wider rectangles indicate exons, whereas narrower regions represent introns or untranslated regions (UTRs). (B) Average DNA methylation levels in promoter regions of these *NgF3H* genes across CG, CHG, and CHH contexts. Data are presented as mean; *n* = 3 biological replicates.

**Figure S15 Methylation patterns and expression levels of the key *NgF3'H1* gene in the DHM biosynthetic pathway.** (A) Base-resolution methylation profiles (IGV snapshots) of *NgF3'H1* across root, stem, and young leaf tissues. The purple bars represent gene structures, and the white arrows indicate the transcriptional direction of the genes. The displayed region encompasses the entire gene locus, from the transcription start site to the transcription termination site. Purple rectangles of different widths represent distinct gene structure regions: wider rectangles indicate exons, whereas narrower regions represent introns or untranslated regions (UTRs). (B) Average DNA methylation levels in the promoter regions of *NgF3'H1* across CG, CHG, and CHH contexts. Data are presented as mean; *n* = 3 biological replicates.

**Figure S16 Methylation patterns and expression levels of the key *NgCHI1* genes in the DHM biosynthetic pathway.** (A) Base-resolution methylation profiles (IGV snapshots) of *NgCHI1* across root, stem, and young leaf tissues. The purple bars represent gene structures, and the white arrows indicate the transcriptional direction of the genes. The displayed region encompasses the entire gene locus, from the transcription start site to the transcription termination site. Purple rectangles of different widths represent distinct gene structure regions: wider rectangles indicate exons, whereas narrower regions represent introns or untranslated regions (UTRs). (B) Average DNA methylation levels in the promoter regions of *NgCHI* across CG, CHG, and CHH contexts. Data are presented as mean; *n* = 3 biological replicates.

**Figure S17 Hypermethylation of competing pathway *NgHCT* genes in young leaves of *N. grossedentata*.** (A) Base-resolution methylation profiles (IGV snapshots) of *NgHCT1***–***NgHCT8* genes, which function in the competing branch pathway diverting substrates from DHM biosynthesis, across root, stem, and young leaf tissues. The purple bars represent gene structures, and the white arrows indicate the transcriptional direction of the genes. The displayed region encompasses the entire gene locus, from the transcription start site to the transcription termination site. Purple rectangles of different widths represent distinct gene structure regions: wider rectangles indicate exons, whereas narrower regions represent introns or untranslated regions (UTRs). (B) Average DNA methylation levels of the *NgHCT* gene family in CG, CHG, and CHH contexts. In contrast to the DHM pathway genes, NgHCTs maintain relatively high methylation levels in young leaves, consistent with their reduced transcript levels and supporting channeling of metabolic flux toward DHM production. Data are presented as mean; *n* = 3 biological replicates.

**Figure S18 Sustained hypermethylation of *NgHCT* genes in the competing metabolic branch of *N. grossedentata*.** (A) Base-resolution methylation profiles (IGV snapshots) of *NgHCT9***–***NgHCT14* genes, which function in the competing branch pathway diverting substrates from DHM biosynthesis, across root, stem, and young leaf tissues. The purple bars represent gene structures, and the white arrows indicate the transcriptional direction of the genes. The displayed region encompasses the entire gene locus, from the transcription start site to the transcription termination site. Purple rectangles of different widths represent distinct gene structure regions: wider rectangles indicate exons, whereas narrower regions represent introns or untranslated regions (UTRs). (B) Average DNA methylation levels of these *NgHCT* genes in CG, CHG, and CHH sequence contexts. The maintenance of high methylation levels in young leaves, in contrast to the hypomethylation of core DHM biosynthetic genes, supports a model where DNA methylation directs metabolic flux toward DHM production. Data are presented as mean; *n* = 3 biological replicates.

**Figure S19 DNA methylation patterns of *NgSTS* genes (*NgSTS1–NgSTS8*) in the competing metabolic branch of *N. grossedentata*.** (A) Base-resolution methylation profiles (IGV snapshots) of *NgSTS1***–***NgSTS8* genes, which function in the competing branch pathway diverting substrates from DHM biosynthesis, across root, stem, and young leaf tissues. The purple bars represent gene structures, and the white arrows indicate the transcriptional direction of the genes. The displayed region encompasses the entire gene locus, from the transcription start site to the transcription termination site. Purple rectangles of different widths represent distinct gene structure regions: wider rectangles indicate exons, whereas narrower regions represent introns or untranslated regions (UTRs). (B) Average DNA methylation levels of these *NgHCT* genes in CG, CHG, and CHH sequence contexts. The maintenance of high methylation levels in young leaves, in contrast to the hypomethylation of core DHM biosynthetic genes, further supports the role of DNA methylation in channeling metabolic flux toward DHM production. Data are presented as mean; *n* = 3 biological replicates.

**Figure S20 DNA methylation patterns of *NgSTS* genes (*NgSTS9–NgSTS16*) in the competing metabolic branch of *N. grossedentata*.** (A) Base-resolution methylation profiles (IGV snapshots) of *NgSTS9–NgSTS16* genes, which function in a competing branch pathway diverting substrates from DHM biosynthesis, across root, stem, and young leaf tissues. The purple bars represent gene structures, and the white arrows indicate the transcriptional direction of the genes. The displayed region encompasses the entire gene locus, from the transcription start site to the transcription termination site. Purple rectangles of different widths represent distinct gene structure regions: wider rectangles indicate exons, whereas narrower regions represent introns or untranslated regions (UTRs). (B) Average DNA methylation levels of these *NgSTS* genes in CG, CHG, and CHH sequence contexts. The maintenance of high methylation levels in young leaves contrasts with the hypomethylation of core DHM biosynthetic genes, supporting the role of DNA methylation in directing metabolic flux. Data are presented as mean; *n* = 3 biological replicates.

**Figure S21 DNA methylation patterns of *NgSTS* genes (*NgSTS17–NgSTS24*) in the competing metabolic branch of *N. grossedentata*.** (A) Base-resolution methylation profiles (IGV snapshots) of *NgSTS17–NgSTS24* genes, which function in a competing branch pathway diverting substrates from DHM biosynthesis, across root, stem, and young leaf tissues. The purple bars represent gene structures, and the white arrows indicate the transcriptional direction of the genes. The displayed region encompasses the entire gene locus, from the transcription start site to the transcription termination site. Purple rectangles of different widths represent distinct gene structure regions: wider rectangles indicate exons, whereas narrower regions represent introns or untranslated regions (UTRs). (B) Average DNA methylation levels of these *NgSTS* genes in CG, CHG, and CHH sequence contexts. The sustained hypermethylation in young leaves further supports the epigenetic channeling of metabolic precursors toward DHM biosynthesis. Data are presented as mean; *n* = 3 biological replicates.

**Figure S22 DNA methylation patterns of *NgSTS* genes (*NgSTS25–NgSTS28*) in the competing metabolic branch of *N. grossedentata*.** (A) Base-resolution methylation profiles (IGV snapshots) of *NgSTS25–NgSTS28* genes, which function in a competing branch pathway diverting substrates from DHM biosynthesis, across root, stem, and young leaf tissues. The purple bars represent gene structures, and the white arrows indicate the transcriptional direction of the genes. The displayed region encompasses the entire gene locus, from the transcription start site to the transcription termination site. Purple rectangles of different widths represent distinct gene structure regions: wider rectangles indicate exons, whereas narrower regions represent introns or untranslated regions (UTRs). (B) Average DNA methylation levels of these *NgSTS* genes in CG, CHG, and CHH sequence contexts. Consistent hypermethylation in young leaves across the *STS* gene family reinforces the model of DNA methylation-mediated regulation of metabolic partitioning. Data are presented as mean; *n* = 3 biological replicates.

**Figure S23 Sustained promoter hypermethylation of downstream *NgFLS* genes in *N. grossedentata*.** (A) Base-resolution methylation profiles (IGV snapshots) of *NgFLS1–NgFLS3* genes, which encode enzymes that compete for shared substrates in the downstream flavonoid pathway, across root, stem, and young leaf tissues. The purple bars represent gene structures, and the white arrows indicate the transcriptional direction of the genes. The displayed region encompasses the entire gene locus, from the transcription start site to the transcription termination site. Purple rectangles of different widths represent distinct gene structure regions: wider rectangles indicate exons, whereas narrower regions represent introns or untranslated regions (UTRs). (B) Average DNA methylation levels in promoter regions of these *NgFLS* genes across CG, CHG, and CHH sequence contexts. The maintenance of high promoter methylation correlates with their suppressed expression, supporting reduced metabolic flux toward competing branches and enhanced channeling toward DHM biosynthesis. Data are presented as mean; *n* = 3 biological replicates.

**Figure S24 Sustained promoter hypermethylation of downstream *NgDFR* genes in *N. grossedentata*.** (A) Base-resolution methylation profiles (IGV snapshots) of *NgDFR1–NgDFR4* genes, which encode enzymes that compete for shared substrates in the downstream flavonoid pathway, across root, stem, and young leaf tissues. The purple bars represent gene structures, and the white arrows indicate the transcriptional direction of the genes. The displayed region encompasses the entire gene locus, from the transcription start site to the transcription termination site. Purple rectangles of different widths represent distinct gene structure regions: wider rectangles indicate exons, whereas narrower regions represent introns or untranslated regions (UTRs). (B) Average DNA methylation levels in promoter regions of these *NgDFR* genes across CG, CHG, and CHH sequence contexts. The maintenance of high promoter methylation correlates with their suppressed expression, supporting reduced metabolic flux toward competing branches and enhanced channeling toward DHM biosynthesis. Data are presented as mean; *n* = 3 biological replicates.

**Figure S25 Negative correlation between promoter methylation and gene expression for DHM biosynthetic genes in *N. grossedentata*.** Scatter plot showing the relationship between the mean promoter methylation level (across all sequence contexts) and log_10_(FPKM + 1) gene expression for all core dihydromyricetin (DHM) biosynthetic genes in young leaves. Each point represents an individual gene, the solid line indicates the linear regression fit. The significant negative correlation supports the role of tissue-specific DNA hypomethylation in facilitating high expression of these genes, thereby promoting DHM accumulation.

**Figure S26 Expression patterns of DNA methyltransferases and demethylases in different tissues of *N. grossedentata*.** Heatmap showing transcript levels (FPKM, *Z*-score normalized) of DNA methyltransferase and demethylase genes in root, stem, and young leaf tissues. Genes highlighted in red indicate demethylases highly expressed or methyltransferases with low expression specifically in young leaves, consistent with the global hypomethylation observed in this tissue.

**Figure S27 Phylogenetic analysis and expression of DNA methyltransferases and demethylases in *N. grossedentata.*** (A) Phylogenetic tree of DNA methyltransferases constructed from conserved domain sequences of *N. grossedentata* (Ng), *Arabidopsis thaliana* (At), *Oryza sativa* (Os), and *Vitis vinifera* (Vv). Bootstrap support values (1000 replicates) are shown at each node as percentages. Red stars mark eight identified Ng methyltransferases: NgAGO4, NgMET1, NgCMT2, NgCMT3, NgDRM1, NgORTH2, NgSUVH4, and NgDDM1. (B) Phylogenetic tree of DNA demethylases from the same four species. Bootstrap support values (1000 replicates) are shown at each node as percentages. Red star marks the identified Ng demethylase NgDML1. For each red-starred gene, expression levels (FPKM, fragments per kilobase of transcript per million mapped fragments) in root, stem, and young leaf tissues are shown as bar plots or annotated values next to the gene name, demonstrating tissue-specific downregulation of methyltransferases and upregulation of NgDML1 in young leaves.

**Figure S28** **Transcription factor landscape associated with DHM biosynthesis in *N. grossedentata.*** Distribution of putative transcription factor families associated with the 28 DHM biosynthetic genes in *N. grossedentata*. Bar plot showing the abundance of predicted TF binding motifs (*e.g.*, MIKC_MADS, BBR-BPC, AP2, and MYB) in the 2 kb promoter regions upstream of 28 structural genes in the DHM pathway. Abbreviation: MYB, v-myb avian myeloblastosis viral oncogene homolog; bHLH, basic helix-loop-helix; AP2, APETALA2; ERF, ETHYLENE RESPONSE FACTOR; RAV, Related to ABI3/VP1; NAC, NAM ATAF1/2 CUC2; WRKY.

**Figure S29 Gene dendrogram and module colors in blocks 1–6 of WGCNA.** The figure shows the hierarchical clustering dendrograms of genes across six blocks, with each block representing a distinct set of genes partitioned by the Weighted Gene Co-Expression Network Analysis (WGCNA) pipeline. The colored rows below each dendrogram indicate the module assignments, where each color represents a specific co-expression module identified by dynamic tree cutting. Modules with similar expression patterns are assigned distinct colors; gray modules represent genes not assigned to any co-expression module.
